# AML associated oncofusion proteins PML-RARA, AML1-ETO and CBFB-MYH11 target RUNX/ETS-factor binding sites to modulate H3ac levels and drive leukemogenesis

**DOI:** 10.18632/oncotarget.14150

**Published:** 2016-12-24

**Authors:** Abhishek A. Singh, Amit Mandoli, Koen H.M. Prange, Marko Laakso, Joost H.A. Martens

**Affiliations:** ^1^ Radboud University, Department of Molecular Biology, Faculty of Science, Nijmegen Centre for Molecular Life Sciences, 6500 HB Nijmegen, The Netherlands; ^2^ Genome Scale Biology Research Program, Research Programs Unit, Faculty of Medicine, University of Helsinki, Helsinki, Finland

**Keywords:** AML, PML-RARA, AML1-ETO, CBFB-MYH11, RUNX1

## Abstract

Chromosomal translocations are one of the hallmarks of acute myeloid leukemia (AML), often leading to gene fusions and expression of an oncofusion protein. Over recent years it has become clear that most of the AML associated oncofusion proteins molecularly adopt distinct mechanisms for inducing leukemogenesis. Still these unique molecular properties of the chimeric proteins converge and give rise to a common pathogenic molecular mechanism. In the present study we compared genome-wide DNA binding and transcriptome data associated with AML1-ETO, CBFB-MYH11 and PML-RARA oncofusion protein expression to identify unique and common features. Our analyses revealed targeting of oncofusion binding sites to RUNX1 and ETS-factor occupied genomic regions. In addition, it revealed a highly comparable global histone acetylation pattern, similar expression of common target genes and related enrichment of several biological pathways critical for maintenance of AML, suggesting oncofusion proteins deregulate common gene programs despite their distinct binding signatures and mechanisms of action.

## INTRODUCTION

Acute myeloid leukemia (AML) is a heterogeneous disease characterized by many genetic variations, including chromosomal translocations. AML is a progressive malignant disease and leads to deranged populations of red blood cells, platelets, and normal white blood cells in bone marrow. AML is the most common form of acute leukemia in adults, is more prevalent in ageing populations and is responsible for ~1% of cancer deaths worldwide [1, 2]. However, AML is a potentially curable disease, although only a minority of patients are cured with current therapies. A large fraction of AMLs is associated with non-random chromosomal translocations [2, 3] that often result in gene rearrangement and expression of an oncofusion protein. Gene rearrangements are believed to provide crucial ground work for cell transformation and initiation of leukemia. Studies have shown that targeting or silencing of these fusion transcripts *in-vitro* leads to reversal of leukemogenesis, decreased proliferation and differentiation [4].

We and others have focused on unraveling molecular aspects of several oncofusion proteins associated with AML, such as AML1-ETO [5, 6, 7, 8], CBFB-MYH11 [9], PML-RARA [10, 11], and FUS-ERG [12]. The AML1-ETO, CBFB-MYH11, and PML-RARA oncofusion protein associated AML-subtypes each account for 5%-10% of all AML cases while FUS-ERG fusions are relatively rare (<1%). The core-binding factor (CBF) oncofusion proteins AML1-ETO and CBFB-MYH11 result from t(8;21) and inv(16) chromosomal rearrangements, while t(15;17) results in expression of PML-RARA. Although these three fusion proteins are generally associated with good prognosis, still 25-58% of relapse incidences are reported in patients with CBF-AMLs [13, 14, 15, 16, 17] and ~10% in patients with t(15;17) [18]. These relapses often cannot be effectively treated with current therapies [19].

Interestingly, genome-wide analysis revealed that molecularly AML1-ETO exhibits properties similar to PML-RARA. Both are believed to exert their functional influence by recruiting repressor complexes containing histone deacetylases to genomic target sites, thereby altering normal chromatin architecture and gene expression [2, 6]. In contrast, genome-wide analysis of CBFB-MYH11, classically believed to be a repressor, has suggested it to be an activator of genes involved in the self-renewal pathway [9].

From the above it is clear that the advent of next generation sequencing has broadened the study of oncofusion proteins from single loci to a genome-wide scale. Together with large cohorts of gene expression data from AML patients that have become available [20, 21, 22], it now provides an opportunity to perform comparative-integrative analysis of these oncofusion proteins to identify exclusive and mutually shared structural and functional features. Here we compared the specific features of PML-RARA, AML1-ETO and CBFB-MYH11 in order to define the common mechanisms in which these proteins are involved in leukemogenesis.

## RESULTS

### AML1-ETO, CBFB-MYH11 and PML-RARA target the RUNX1/ETS gene program

**Figure 1:**
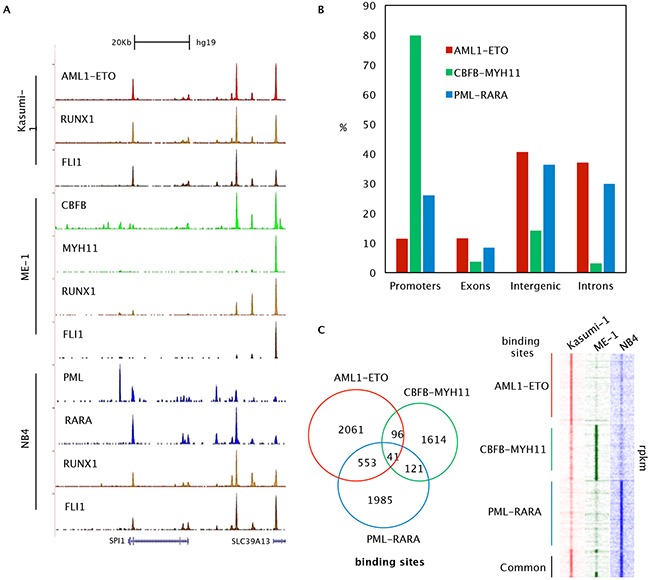
Genome-wide binding features of oncofusion proteins **A**. Overview of the SPI1 and SLC39A13 AML1-ETO, CBFB-MYH11, PML-RARA, RUNX1 and FLI binding sites in Kasumi-1, ME-1 and NB4 cells. **B**. Distribution of the AML1-ETO, CBFB-MYH11 and PML-RARA binding site locations relative to RefSeq genes. Locations of binding sites are divided in promoter (-500 bp to the transcription start site), exon, intron and intergenic (everything else). **C**. Left: Venn diagram representing the overlap of AML1-ETO, CBFB-MYH11 and PML-RARA binding sites in Kasumi-1, ME-1 and NB4 cells. Right: Heatmap displaying tag densities in unique and shared binding sites of AML1-ETO, CBFB-MYH11 and PML-RARA oncofusion proteins.

To identify the common binding and gene program of oncofusion proteins associated with AML we used previously identified target regions of PML-RARA in the t(15;17) NB4 cell line [10], AML1-ETO in the t(8;21) Kasumi-1 cell line [6] and CBFB-MYH11 in the inv(16) ME-1 cell line [9] for further analysis (Figure 1A). This revealed that the two core binding factor-oncofusion proteins, AML1-ETO and CBFB-MYH11 differ significantly in their genomic distribution (Figure 1B), with AML1-ETO preferentially targeting distal elements and CBFB-MYH11 mostly promoter bound. In contrast, PML-RARA, which does not represent a core binding factor is similar in its targeting as AML1-ETO, mostly locating to distal sites (Figure 1B). In line, PML-RARA and AML1-ETO share more binding sites (~20%) as compared to CBFB-MYH11 and AML1-ETO (~5%), and CBFB-MYH11 and PML-RARA (~6%) (Figure 1C).

**Figure 2:**
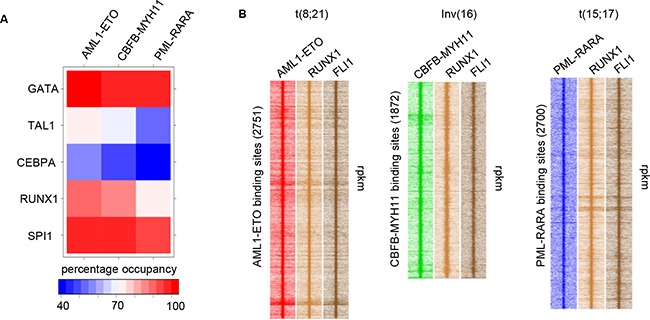
Oncofusion binding sites are demarcated by RUNX1 and members of the ETS factor family **A**. Plot depicting percentages of AML1-ETO, CBFB-MYH11 and PML-RARA binding sites harboring consensus sequences of transcription factors. **B**. Heatmap displaying tag densities at AML1-ETO, CBFB-MYH11 and PML-RARA binding in Kasumi-1, ME-1 and NB4 cells, respectively.

To investigate whether the fusion proteins target a common transcription factor network we performed motif enrichment analysis on their binding sites, using weight matrices of AML1/RUNX1, C/EBPA, the ETS factor SPI1, GATA and TAL1, all transcription factors reported to be mutated in acute leukemias [23]. This revealed significant enrichment for AML1/RUNX1, SPI1, and GATA motifs at all sites, while TAL1 is more specific for core binding factors and enrichment for C/EBPA motifs is comparatively reduced at all sites (Figure 2A). Previous studies have shown co-occupancy of RUNX1 and the ETS factors FLI1/ERG at AML1-ETO and CBFB-MYH11 binding sites [6, 9]. Here, our analysis suggests also enrichment for RUNX1 and ETS factors at binding sites of PML-RARA. Indeed, examining RUNX1 and FLI1 enrichment at PML-RARA oncofusion protein binding sites confirmed enrichment of both (Figure 1A, 2B). Together these results suggest a role for RUNX1 and members of the ETS family in modulating oncofusion protein binding and a possible role in regulating gene expression as suggested in previous studies [6, 9].

### Oncofusion proteins AML1-ETO, CBFB-MYH11 and PML-RARA target similar biological pathways

**Figure 3:**
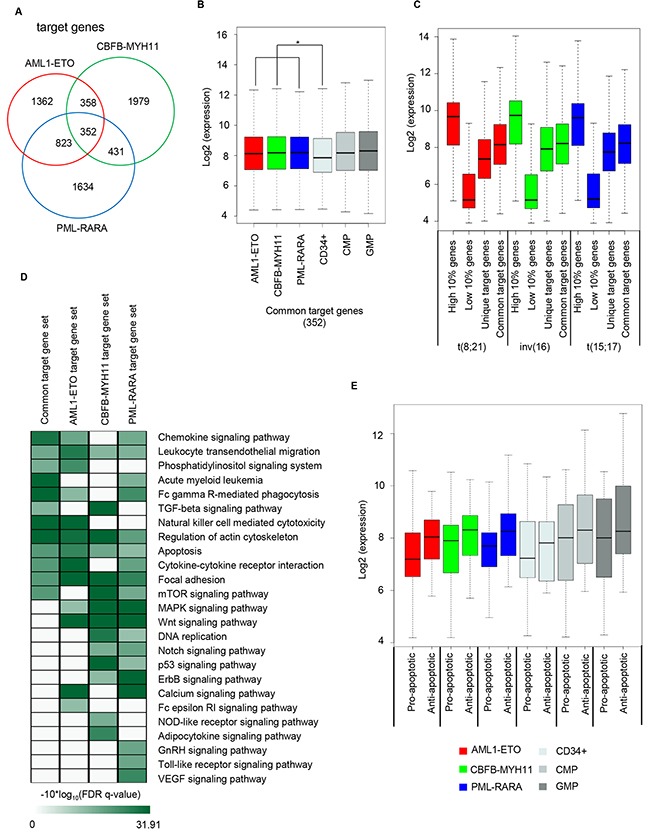
Expression of oncofusion target genes **A**. Venn diagram representing overlap between the target genes of AML1-ETO, CBFB-MYH11 and PML-RARA. **B**. Higher expression of common target genes of AML1-ETO, CBFB-MYH11 and PML-RARA is observed in t(8;21), inv(16) and t(15;17) AML sub-types as compared to normal CD34^+^ (*p-value <0.05). While the expression in other cell types (CMP and GMP) doesn't exhibit a significant change in expression. **C**. Comparison of expression of he 10% highest expressed genes, the 10% lowest expressed genes, unique target and common target genes of AML1-ETO, CBFB-MYH11 and PML-RARA in t(8;21), inv(16) and t(15;17) AML subtypes. **D**. Heatmap displaying enrichment of biological pathways. The common, AML1-ETO, CBFB-MYH11 and PML-RARA gene set enrich identical as well as distinct pathways, suggesting similar pathways are enriched from distinct gene sets. **E**. Expression of apoptotic and anti-apoptotic genes in t(8;21), inv(16), t(15;17), CD34+, CMP and GMP cells.

Progression of leukemia is not a consequence of a single deregulated pathway but is believed to be a synergistic impact of a wide range of misregulated pathways including cell cycle, differentiation, signaling, apoptosis and self-renewal pathways [24, 25, 26]. To identify the target gene programs of the oncofusion proteins we assigned binding sites to nearest genes within a window of 25 kb for further analysis. This revealed that many target genes are shared between the three oncofusion proteins with AML1-ETO and PML-RARA sharing a maximum number of 1175 (~40%) genes (Figure 3A), in line with their higher overlap of binding sites. Examining all 3 oncofusion proteins revealed 352 (~11%) genes commonly targeted, suggesting that despite occupying different genomic regions (Figure 1B), still many of the same genes are targeted. Furthermore, on investigating the set of genes differentially expressed upon AML1-ETO knockdown in t(8;21) cells [5] in inv(16) AML cells before and after knockdown of CBFB-MYH11 revealed differential expression of 45% of genes (Supplementary Figure 1A and Supplementary Table 1). These results suggest that a large fraction of the target genes is indeed shared between the oncofusion proteins and likely involved in regulation of leukemogenesis.

Interestingly, this common target gene set exhibits significant higher expression across all fusion protein expressing AML subtypes as compared to normal CD34+ cells (Figure 3B), but similar to CMP and GMP progenitor populations, suggesting these represent the differentiation stage in which the AMLs are blocked. Comparison with the unique target gene set for each oncofusion protein revealed increased expression of the common gene set in all three AMLs (Figure 3C). Functional annotation of the common and unique target gene set revealed involvement of genes in many pathways, including acute myeloid leukemia, apoptosis and the TGF-β signaling pathway. In addition, enrichment of identical pathways through distinct gene sets was observed (Figure 3D). Higher expression of the genes associated with these pathways (Figure 3B & 3C) can have different functional effects as exemplified by the apoptosis pathway, which is enriched by the common as well as unique target gene set of all three oncofusion proteins (Figure 3D). As the apoptosis pathway contains both anti- (e.g. BCL2) as well as pro- (e.g. BID) -apoptotic genes [27] we examined expression of both sets revealing that generally anti-apoptotic genes are higher expressed in AML, but also in normal progenitors (Figure 3E). Increased expression of anti-apoptotic genes in AML is in line with the presence of the ‘prevention of cell death’ hallmark of cancer [28] and decreased apoptosis in oncofusion protein expressing AML cells.

### AML subtype specific expression of genes

**Figure 4:**
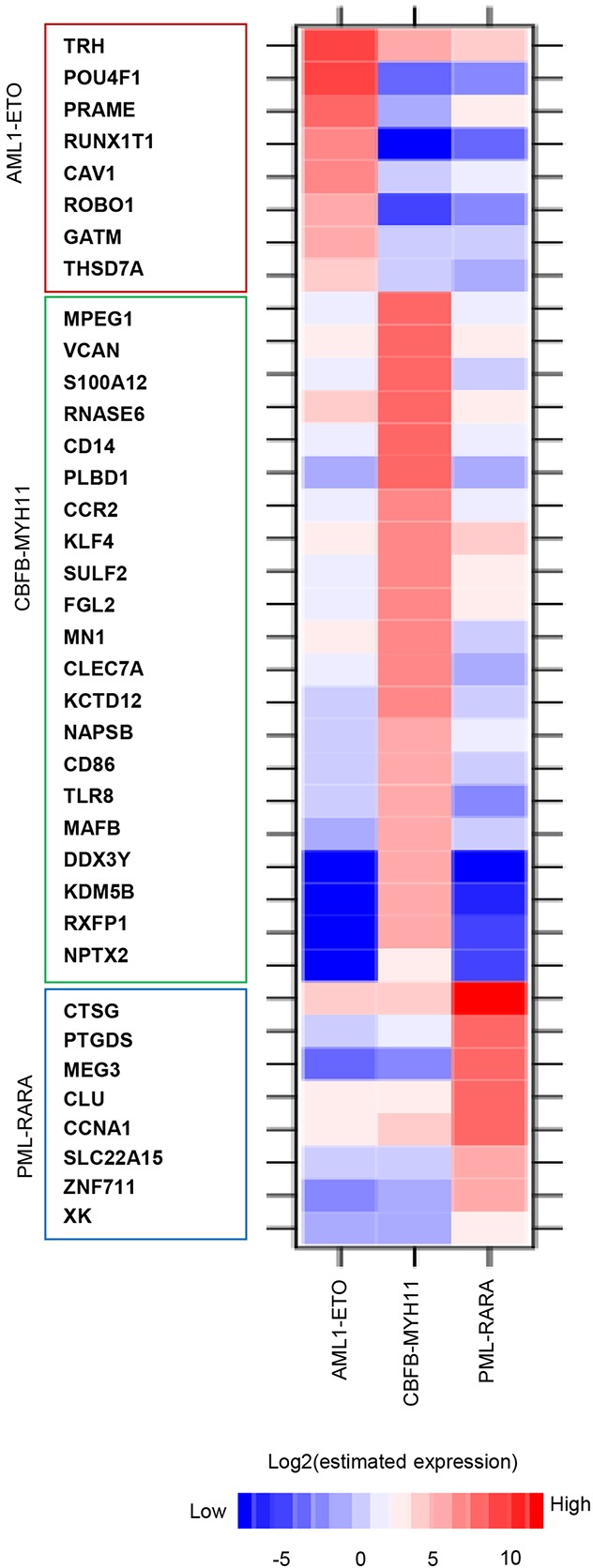
AML signature gene identification Heatmap displaying expression levels of signature genes for t(8;21), inv(16) and t(15;17) AML identified in two cohort studies [21, 22].

Despite targeting a common gene program a subset of genes is controlled by only one of the three oncofusion proteins (Figure 3A), suggesting the presence of AML subtype specific gene signatures. To identify specific gene expression signatures for each subtype of AML we analyzed gene expression in primary AMLs expressing AML1-ETO, CBFB-MYH11 and PML-RARA [20, 21]. This led to the identification of signature genes for the three subtypes, including POU4F1 and PRAME genes in t(8;21) translocation, VCAN and S100A12 in inv(16) and CTSG and PTGDS in t(15;17) AMLs. Comparing these data to more recent RNA-seq analysis [22] confirmed these observations (Figure 4), suggesting the identification of a stable AML subtype specific gene signature that potentially can be used as biomarker.

### AML1-ETO, CBFB-MYH11 and PML-RARA oncofusion proteins alter the acetylome

Acetylation and deacetylation of histones catalyzed by histone acetyltransferases (HATs) and histone deacetylases (HDACs), respectively, are two of the several mechanisms controlling the complex process of transcription. HATs and HDACs play opposite roles in gene regulation by modulating chromatin structure and activity of certain transcription factors by differential acetylation [29].

In cancerous cells acetylation levels are believed to be imbalanced and influencing expression levels of tumor suppressor genes and proto-oncogenes. Hyper-acetylated promoters of proto-oncogenes are suggested to increase expression of these genes and turn them into oncogenes. In contrast, hypo-acetylation of promoters of tumor suppressor genes invariably silences them [30]. Deregulation of target genes by (de)acetylation seems a common mechanism for oncofusion proteins. Originally only associated with recruitment of HDACs, the oncofusion proteins AML1-ETO, PML-RARA and CBFB-MYH11 have recently been associated both with HAT and HDAC protein complex binding to balance histone acetylation output [6, 9, 10, 31, 32].

**Figure 5:**
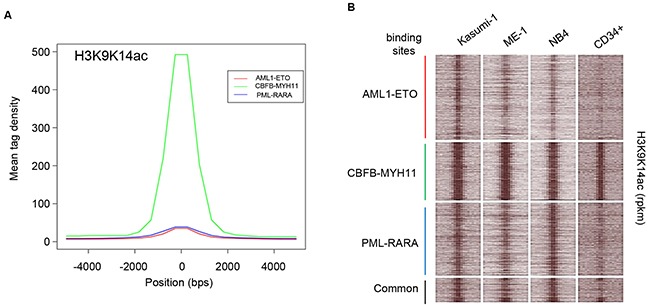
Histone H3 acetylation at AML1-ETO, CBFB-MYH11 and PML-RARA binding sites **A**. H3 acetylation profile at AML1-ETO binding sites in Kasumi-1, at CBFB-MYH11 binding sites in ME-1 and at PML-RARA binding sites in NB4 cells. **B**. Heatmap displaying tag densities of H3K9K14ac in unique and shared binding sites of AML1-ETO, CBFB-MYH11 and PML-RARA oncofusion proteins.

Here, we compared binding sites of the three oncofusion proteins for H3 acetylation (H3K9K14ac) enrichment and observed high levels of acetylation at CBFB-MYH11 binding sites as compared to AML1-ETO and PML-RARA (Figure 5A). This is likely related to the differences in mechanistic properties, with CBFB-MYH11 mostly binding, and potentially stabilizing, active promoters while AML1-ETO and PML-RARA bind and stabilize inactive enhancers. Interestingly, on examining global acetylation of CBFB-MYH11 defined regions in t(8;21) and t(15;17) cells we observe similar levels of increased acetylation (Figure 5B), while vice versa, comparatively reduced levels of acetylation at PML-RARA and AML1-ETO binding sites are also observed in inv(16) cells. Furthermore, acetylation levels at these oncofusion protein binding sites seem further reduced in normal CD34+ cells (Figure 5B, Supplementary Figure 1B), suggesting a specific acetylation signature for these leukemic cell types. Together these results suggest that despite oncofusion protein binding to different genomic regions, the resulting acetylome is to a large extend similarly organized in these AML cells.

## DISCUSSION

Many breakpoints involved in specific chromosomal translocations have been cloned over the years. In most cases, however, the role of the chimeric oncofusion proteins in tumorigenesis has not been elucidated. In the case of AML, our analysis of PML-RARA, AML1-ETO, and CBFB-MYH11 were among the first to report on the genome-wide actions of oncofusion proteins. These genome-wide studies focused on revealing various structural and functional aspects of the oncofusion proteins like composition of the protein complexes formed by oncofusion proteins, the effects on the epigenetic landscape, the enriched pathways and deregulated gene networks. None of these studies carried out an integrative comprehensive analysis on the genome-wide maps of different oncofusion proteins and identify unique and mutually shared features between them.

Despite the suggested differences between these oncofusion proteins, the data presented here reveals the binding sites of all three are demarcated by RUNX1 and ETS factors indicating the existence of a common functional RUNX1/ETS-factor module that is targeted in oncofusion mediated leukemogenesis. Similarly, the commonly targeted genes by all three oncofusion proteins show higher expression patterns in AML-subtypes as compared to CD34+ cells and enrich biological pathways crucial for leukemogenesis. Amongst these pathways are several signaling modules which likely stimulate cell proliferation, such as the TGFB signaling pathway [33]. In addition, all are enriched for genes involved in regulation of apoptosis, in particular anti-apoptotic genes which are higher expressed to prevent cell death. Together these data indicate that the distinct molecular properties of these oncofusion proteins converge on common mechanisms to transform cells.

**Figure 6:**
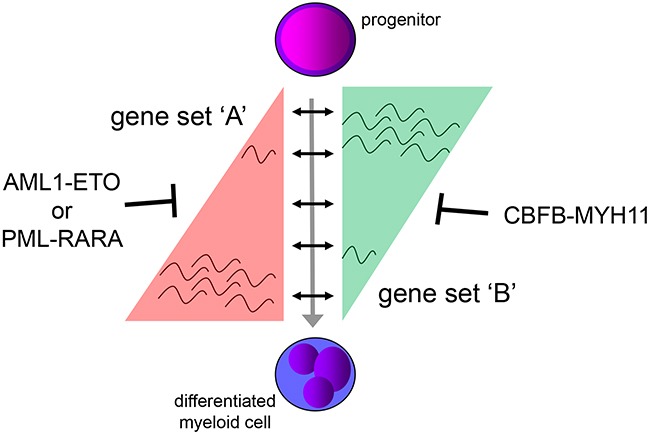
Proposed mechanism for expression deregulation by AML1-ETO, CBFB-MYH11 and PML-RARA The AML1-ETO and PML-RARA on one side and CBFB-MYH11 on the other side modulate gene expression using different mechanisms, but genes are interdependent. A gene set ‘A’ cannot be activated when a gene set ‘B’ is repressed and vice versa. By blocking activation of gene set ‘A’ reduced expression of gene set ‘B’ will be prevented and cells will be locked in a progenitor state (proposed for AML1-ETO and PML-RARA). A similar block can be created for gene set ‘A’ by maintaining activity of gene set ‘B’ (proposed for CBFB-MYH11).

Although utilizing distinct mechanisms to regulate the local epigenetic environment, with AML1-ETO and PML-RARA suggested to reduce acetylation (and expression of genes) [5, 6, 10, 32] and CBFB-MYH11 to increase acetylation [9], the overall acetylome of all oncofusion protein binding sites seems very similar (Figure 5B). Despite different epigenetic and transcriptional effects at an individual gene level, these results suggest that each oncofusion protein fixes cells in a common ‘average leukemic’ state, with similar pathways activated and repressed. We propose that the combined set of all oncofusion binding sites represents interdependent regions that need to be switched on/off during hematopoietic differentiation (Figure 6). Although AML1-ETO and PML-RARA on one side and CBFB-MYH11 on the other side modulate gene expression through distinct mechanisms, the interdependencies still results in common global profiles in this model. Either gene set ‘A’ can be increased, resulting due to interdependency in decreased activity of gene set ‘B’ and subsequent differentiation, or gene set ‘B’ can be decreased, which results in increased expression of gene set ‘A’ and a similar expression profile. We propose that oncofusion proteins use this interdependency to lock differentiation pathways. Blocking gene set ‘A’ from becoming activated (for example as is the proposed mechanism for AML1-ETO and PML-RARA) will simultaneously prevent decreased expression of gene set ‘B’ and will lock the cells in a progenitor state. Similarly, maintaining activity of gene set ‘B’ (as being proposed for CBFB-MYH11) will prevent increased expression of gene set ‘A’ and results in a similar block. This ‘gene set’ lock might be further strengthened by additional co-occurring mutations.

Together, the commonalities discussed in this study might present novel entry points for further treatment of AML patients with these translocations, for example by targeting the RUNX1/ETS-factor interaction, disruption of the associated complex, or use of specific compounds that target one of the common pathways altered by the fusion proteins, such as BCL2 inhibitors [34, 35] to block the activation of the anti-apoptosis program. Finally, the common acetylation signature present in these cells might be targeted by specific drugs. While HDACi's would be an obvious choice [36], the general toxicity invoked by these hyper-acetylating compounds makes them less suitable [37]. Using the alternative strategy of blocking acetylation to induce hypoacetylation, for example by iBETs or HATi might prove to be a better strategy in the long run.

## EXPERIMENTAL PROCEDURE

### Chromatin immunoprecipitation (ChIP)

Cell line were cross-linked with 1% formaldehyde for 20 min at room temperature, quenched with 0.125 M glycine and washed. Sonicated chromatin (Bioruptor, Diagenode, Liege, Belgium) was centrifuged at maximum speed for 10 min and then incubated with specific antibodies. Beads were washed sequentially with four different wash buffers and chromatin was eluted from the beads. Protein–DNA crosslinks were reversed, after which DNA was isolated and used for quantitative PCR or sequencing analysis.

### Illumina high-throughput sequencing

End repair was performed using the precipitated DNA of ~6 million cells (3-4 pooled biological replicas) using Klenow and T4 polynucleotide kinase (T4 PNK). A 3′ protruding A base was generated using Taq polymerase and adapters were ligated. The DNA was loaded on gel and a band corresponding to ~300 bp (ChIP fragment + adapters) was collected. The DNA was isolated, amplified by PCR and used for cluster generation on the Illumina HiSeq genome analyzer. The 35–45 bp tags were mapped to the reference human genome using the Burrows-Wheeler Alignment Tool (BWA) or eland program allowing one mismatch. For each base pair in the genome, the number of overlapping sequence reads was determined, averaged over a 10 bp window and visualized in the UCSC genome browser (http://genome.ucsc.edu). ChIP-seq data can be downloaded from Gene Expression Omnibus accession numbers GSE46044, GSE23730, GSE30254, GSE18886 and GSE81992.

### Bioinformatic analysis

#### DNA binding & expression data

Raw ChIP-seq data files for AML1-ETO, CBFB-MYH11 and PML-RARA oncofusion proteins, transcription factors RUNX1 and FLI1, and acetylation (H3K9K14ac) profiles in Kasumi-1, ME-1 and NB4 (GSE23730, GSE76464, GSE46044, GSE18886) cells were downloaded and mapped to hg19 using bwa [38]. Expression data sets were obtained from cohort studies in AML patients by Verhaak et al., 2009 (GSE6891) [21] and from The Cancer Genome Atlas Research Network (TCGA; http://cancergenome.nih.gov/) [22].

#### Peak extraction

Published DNA binding sites associated with AML1-ETO [6], CBFB-MYH11 [9] and PML-RARA [10] oncofusion proteins were converted to hg19 (liftOver; https://genome.ucsc.edu/cgi-bin/hgLiftOver).

#### Tag counting

Tags within a given region were counted and adjusted to represent the number of tags within 1Kb regions. Subsequently the percentage of these tags as a measure of the total number of tags of the sample was calculated and displayed as heatmaps and/or line graph.

#### Peak distribution

To determine genomic locations of binding sites, the peak files were analyzed using a script that annotates binding sites according to all RefSeq genes. With this script every binding site is annotated either as promoter (-500 bp to the Transcription Start Site), exon, intron or intergenic (everything else).

#### Motif analysis

For motif analysis of AML1-ETO, CBFB-MYH11 and PML-RARA binding sites, we used GimmeMotifs [39].

#### Enrichment analysis

Pathway and gene ontology (biological process) enrichment analysis was performed using molecular signature database of Gene Set Enrichment Analysis (GSEA; http://software.broadinstitute.org/gsea/) [40].

#### Identification of potential signature genes

The expression data associated with AML subtypes [21, 22] was used to identify potential signature genes for each of the subtypes. We used microarray expression data [21] as our preliminary set to identify genes that were upregulated at a FC ≥ 8 in only one of the subtypes. This preliminary list of potential signature genes was screened against a recent cohort study [22] conducted on a next-generation sequencing platform. The genes passing both these filters successfully were marked as potential signatures for the associated AML subtypes.

## SUPPLEMENTARY MATERIALS FIGURES AND TABLES


