# Supplementary material for: AML associated oncofusion proteins PML-RARA, AML1-ETO and CBFB-MYH11 target RUNX/ETS-factor binding sites to modulate H3ac levels and drive leukemogenesis
Source: Oncotarget. 2016 Dec 24;8(8):12855–65. doi: 10.18632/oncotarget.14150 (PMC5355061; doi:10.18632/oncotarget.14150)
Supplement: Supplementary file 1 [file oncotarget-08-12855-s001.pdf]

AML associated oncofusion proteins PML-RARA, AML1-ETO and CBFB-MYH11 target RUNX/ETS-factor binding sites to modulate H3ac levels and drive leukemogenesis

SUPPLEMENTARY FIGURE AND TABLE

A

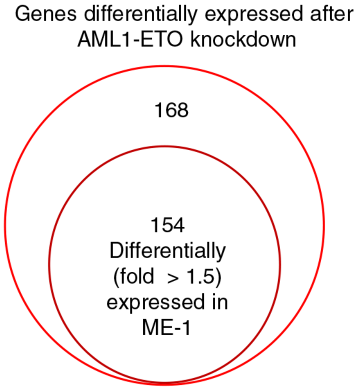

B

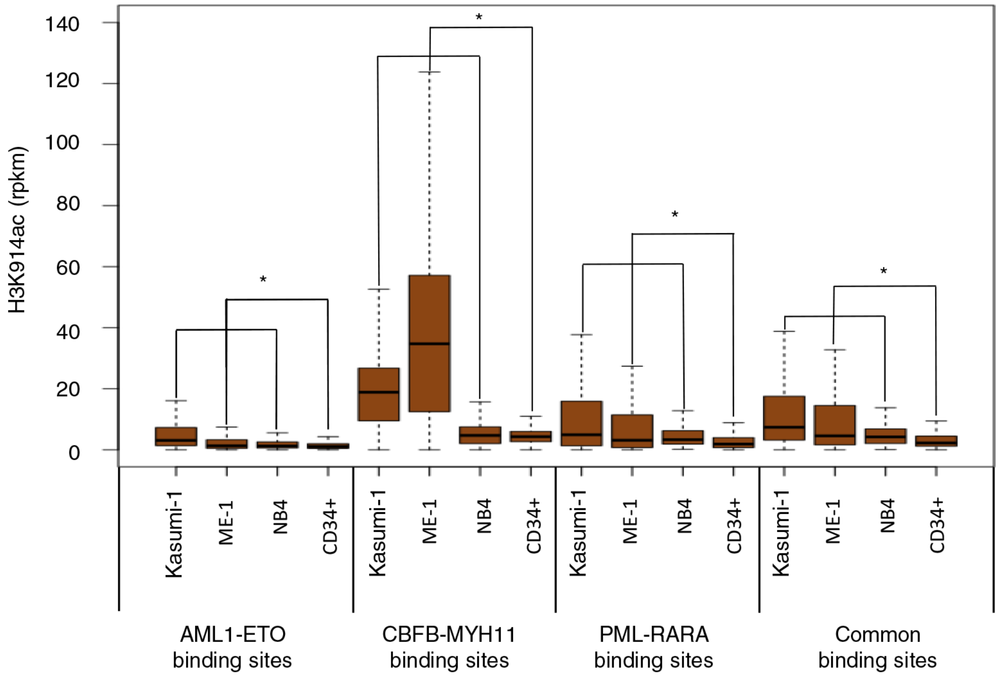

Supplementary Figure 1: A. Venn diagram showing that154 out of 322 genes differentially expressed after AML1-ETO knockdown in Kasumi-1 cells are also differentially expressed in ME-1 cells upon CBFB-MYH11 knockdown. **B.** Oncofusion binding sites significantly differ in acetylation levels (*p-value<0.01) between leukemic cells and normal CD34+ cells.

Supplementary Table 1: Expression levels of the gene set identified after AML1-ETO knockdown in Kasumi-1 cells [5] before and after knockdown of CBFB-MYH11 in ME-1 cells

See Supplementary File 1
